# Identifying factors related to delayed neck metastasis after surgical treatment in patients with oral squamous cell carcinoma

**DOI:** 10.1186/s40902-024-00430-z

**Published:** 2024-06-17

**Authors:** Sang-Min Lee, Hyosik Kim, Kang-Min Ahn

**Affiliations:** Department of Oral and Maxillofacial Surgery, Asan Medical Center, College of Medicine, University of Ulsan, Seoul, South Korea

**Keywords:** Oral squamous cell carcinoma, Delayed neck metastasis, Cumulative survival rate

## Abstract

**Background:**

General treatment of oral squamous cell carcinoma (OSCC) is surgical treatment with or without neck dissection. Although the incidence of delayed neck metastasis is rare, it may occur after the surgery and is known to be the most important factor in the prognosis. The purpose of is study is to evaluate the clinical and histopathological factors associated with delayed neck metastasis case among patients.

**Methods:**

A total of 195 patients who underwent surgical treatment for OSCC from 2016 to 2022 were investigated. Among them, delayed neck metastasis (DNM) was analyzed. The criterion for delayed neck metastasis was a newly developed neck lesion after the primary operation without neck dissection in cN0 necks. To identify the correlation between prognostic factors and the incidence of delayed neck metastasis, *χ*^2^ analysis with phi correlation and Cramer’s *V* test was performed. Cumulative survival rates (CRS) were compared between the groups with the incidence of DNM and without DNM. Also, the log rank test for CSR and Cox proportional hazard model was analyzed to estimate the significance of the CSR and confirm the correlations between prognostic factors and DNM.

**Result:**

Among 195 patients, 14 were discovered to have DNM. The primary tumor locations were the tongue (*n* = 5), floor of the mouth (*n* = 2), mandibular gingiva (*n* = 1), maxillary gingiva (*n* = 4), retromolartrigone (*n* = 1), and buccal mucosa (*n* = 2) each. The cases consisted of TNM stage I (*n* = 1), stage II (*n* = 3), stage III (*n* = 3), and stage IV (*n* = 8), respectively. The result of the *χ*^2^ analysis identified a correlation between positive neck (*p* = 0.01), depth of invasion (*p* = 0.09), radiation therapy (*p* = 0.003), and DNM. Groups without DNM showed better prognosis compared to groups with DNM. Regarding positive neck, depth of invasion, and radiation therapy, only depth of invasion showed significance in CSR analysis.

**Conclusion:**

DNM after surgical treatment of OSCC is a rare event, and few were found in a review of the literature. Also, many prognostic factors have been suggested but controversial. However, in our study, some prognostic factors have been identified to have a significant correlation with the incidence of DNM, and analysis of such factors provides important information predicting neck metastasis and the prognosis.

## Background

The rate of neck metastases of oral squamous cell carcinoma (OSCC) is known to be about 20–30% [1–3]. However, delayed neck metastasis (DNM) of OSCC is known to be a rare event accounting for 3 ~ 9% of all head and neck cancers [4]. Also, the issue of DNM received few attentions in the previous studies, and relatively few reports were found in a review of the literature [5–8]. Furthermore, DNM on the contralateral side is rarer than the ipsilateral side. The positive lymph nodes on the ipsilateral side of the neck at the time of diagnosis are considered to be the most related factor in the development of metastasis on the contralateral side [9, 10]. The primary prognostic factor for head and neck cancer is whether cervical lymph node metastases have occurred or not [11, 12]. Therefore, the treatment of the disease relies heavily on the proper management of the lymph nodes after primary surgery [13]. According to previous studies, the incidence of neck metastasis might vary from 15 to 60% depending on several prognostic factors [14–16]. Such factors include tumor stage (TNM stage), histological differentiation and grading, depth of tumor, perineural invasion, and lymphovascular invasion [17, 18]. Although such factors were not evaluated precisely according to previous studies, they may be significant causes of delayed neck metastasis [19].

The most precise technique for determination and diagnosis of the lymph node status is the surgical exploration during the procedure of elective or therapeutic neck dissection (ND) and histopathologic evaluation of removed neck nodes [20]. The treatment procedure for neck lymph nodes depends on the treatment plan of the primary cancer and the clinical evaluation of cervical lymph node metastasis [21]. The procedure of elective neck dissection is acknowledged if the risk of occult metastasis is higher than 15–20% or when readings of magnetic resonance imaging show positive cervical lymph node metastasis [21, 22]. The purpose of the present study was to evaluate the incidence of DNM in patients with OSCC and analyze the prognostic factors of OSCC.

## Methods

### Patients

**Fig. 1:**
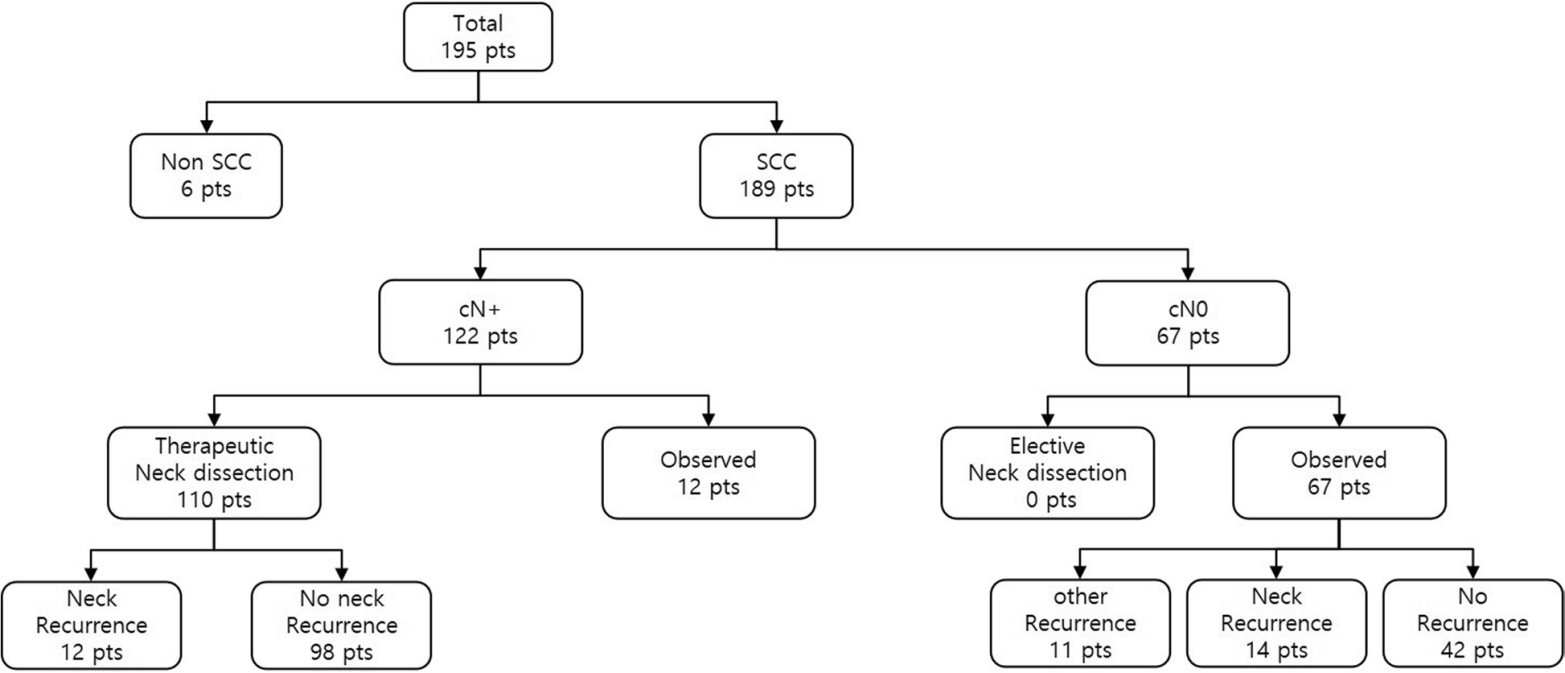
The inclusion criteria of patients in the study

A retrospective study included clinical data of patients with OSCC who underwent surgical treatment from 2006 to 2022 by a single surgeon at the Department of Oral and Maxillofacial Surgery. The patients were selected by strict inclusion and exclusion criteria. A total of 195 patients were investigated. Patients who turned out to be diagnosed with other diseases after primary resection were excluded (*n* = 6). Patients who were diagnosed with positive neck in clinical examinations were excluded (*n* = 122). Patients with local recurrence, regional recurrence, distant recurrence, and second primary OSCC were excluded (*n* = 11). The inclusion criteria contained patients with cN0 neck patients who have not undergone neck dissection during the initial surgery (*n* = 56). In our study, DNM was defined as the incidence of newly developed metastatic neck lesion after complete resection of the primary tumor and without evidence of residual lesion in cN0 neck patients. The inclusion criteria are described in Fig. 1.

### Diagnosis and surgical procedures

Patients were staged via manual examination, result of ultrasonography or computed tomography (CT), and magnetic resonance (MRI). Therapeutic neck dissection was performed if the result of magnetic resonance imaging showed positive neck lymph node metastasis. In general, in case of advanced OSCC at stage III or beyond, elective neck dissection could be performed as a preventive method. However, in our study, if reconstruction using free flap was not deemed necessary due to the relatively small size of the tumor or if the patient’s overall condition was not suitable for surgery due to old age, we adopted the approach of only performing mass excision followed by direct closure rather than including neck dissection at the time of the initial surgery.

The primary surgery was performed with free flap reconstruction when the defect after radical resection of OSCC is unable to close directly. The surgery contained mass excision with or without neck dissection. The resection margin of the mass was set 1 to 1.5 cm safety margin. A frozen biopsy was done during the surgery, and further resection with an additional frozen biopsy was performed until a negative resection margin was confirmed. If necessary, free flap reconstruction was performed by the same surgeon with methods of radial forearm free flap, fibular free flap, and latissimus dorsi free flap. Postoperative radiotherapy was decided depending on the results of the biopsy including positive or close resection margin.

### Statistical methods

Gender, age (below 65, 65 and above), clinical staging according to TNM classification (I, II, III, IV), primary tumor site (tongue, floor of the mouth, mandibular gingiva, maxillary gingiva, mandibular vestibule, maxillary vestibule, retromolartrigone, hard palate, soft palate, buccal mucosa, PIOS), grade of histologic differentiation (well differentiated, moderated differentiated, poorly differentiated), depth of invasion (under 5 mm, 5 mm and over), perineural invasion (positive, negative), and lymphovascular invasion (positive, negative) were analyzed as critical factors for incidence of delayed neck metastasis. In the case of the primary tumor site, the operational definition was established to specify the precise locations of the tumor. The term “gingiva” was defined to include the attached gingiva, and for “vestibule,” it was defined to encompass the connective tissue beyond the mucogingival junction.

Univariate analysis with the *χ*^2^ test with phi correlation and Cramer’s *V* test was performed to evaluate the correlation between neck metastasis and prognostic factors. The level of significance was set as *p* < 0.05. All factors analyzed in the study are described in Table 1. In addition, the Kaplan–Meier methods with the log rank test were performed to compare the cumulative survival rate (CSR) between patients with and without DNM, as well as within DNM patients based on prognostic factors which turned out to be significantly correlated with the incidence of the disease. Also, the Cox proportional hazard model was analyzed to confirm the correlation of prognostic factors and DNM. All statistical analyses were performed by using the IBM SPSS for Windows (ver. 22.0).

**Table 1:** Comparison of cumulative survival rates between patients with incidence of delayed neck metastasis and without delayed neck metastasis (disease-free)

Factor	No. of cases (%)	No. of delayed neck metastasis (%)	*χ*^2^ test with phi correlation, Cramer’s *V* test *p*-value
Gender			
Male	40 (71.43)	9 (64.29)	0.495
Female	16 (28.57)	5 (35.71)	
Age (years)			
< 65	26 (46.43)	7 (50.00)	0.533
≥ 65	30 (53.57)	7 (50.00)	
T-stage			
T1	17 (30.36)	1 (7.14)	0.03^*^
T2	13 (23.21)	3 (21.43)	
T3	6 (10.71)	3 (21.43)	
T4	20 (35.71)	7 (50.00)	
DOI			
< 5	23 (41.07)	3 (21.43)	0.0085^*^
≥ 5	33 (58.93)	11 (78.57)	
PNI			
Yes	3 (5.36)	1 (7.14)	0.732
No	53 (94.64)	13 (92.86)	
LVI			
Yes	6 (10.71)	2 (14.29)	0.618
No	50 (89.29)	12 (85.71)	
Histologic grade			
Well differentiated	26 (46.43)	4 (28.57)	0.122
Moderate differentiated	30 (53.57)	10 (71.43)	
Poorly differentiated	0 (0)	0 (0)	
RT			
Yes	23 (41.07)	10 (71.43)	0.008^*^
No	33 (58.93)	4 (28.57)	
Site			
Lip	1 (1.79)	0 (0)	0.669
Tongue	15 (26.79)	5 (35.71)	
FOM	3 (5.36)	2 (14.29)	
Mn gingiva	10 (17.86)	1 (7.14)	
Mx gingiva	12 (21.43)	3 (21.43)	
Mn vestibule	1 (1.79)	0 (0.00)	
Mx vestibule	0 (0.00)	0 (0.00)	
RMT	6 (10.71)	1 (7.14)	
Hard palate	0 (0.00)	0 (0.00)	
Soft palate	1 (1.79)	0 (0.00)	
BM	7 (12.50)	2 (14.29)	
PIOS	0 (0.00)	0 (0.00)	
Total	56 (100.00)	14 (100.00)	

*DOI* Depth of invasion, *PNI* Perineural invasion, *LVI* Lymphovascular invasion, *RT* Radiotherapy, *FOM* Floor of the mouth, *Mn* Mandible, *Mx* Maxilla, *RMT* Retromolar trigone, *BM* Buccal mucosa, *PIOS* Primary intraosseous squamous cell carcinoma

^*^P < 0.05

## Result

A total of 195 patients underwent surgery, and among them, 56 patients were identified to meet the inclusion criteria. Among them, 14 patients had an incidence of DNM (Table 2). The age ranged from 37 to 90 years with a mean age of 66.5 ± 13.78 years. The patients consisted of 40 males (71.43%) and 16 female (28.57%). Out of these, 18 patients died during the follow-up period. The range of the follow-up period before the incidence of neck metastasis for the 14 patients was 1 to 68 months, with an average of 10.79 ± 16.17 months. The average duration of survival was 27 ± 19.99 months (range, 7 to 78 months). The chi-square test through cross-analysis revealed that age (younger than 65 vs 65 and older) and gender did not show any significant results in relation to the occurrence of DNM.

**Table 2:** Follow-up periods and occurrence of delayed neck metastasis

Gender	Age	Primary site of cancer	Occurrence of delayed neck metastasis (months after primary surgery)	Survival period (months)	Survival
Female	65	Buccal mucosa	68	78	Yes
Male	81	Tongue	6	14	No
Female	85	Tongue	6	49	No
Male	60	Buccal mucosa	5	51	Yes
Male	60	Maxillary gingiva	9	9	No
Female	37	Tongue	8	13	No
Male	82	Retromolar trigone	1	36	No
Female	63	Mandibular gingiva	4	33	Yes
Female	49	Floor of mouth	15	29	Yes
Male	70	Tongue	5	20	Yes
Male	78	Maxillary gingiva	7	7	No
Male	80	Maxillary gingiva	4	6	No
Male	62	Floor of mouth	8	14	Yes
Male	57	Tongue	5	19	No

Out of the total 14 cases, 1 was diagnosed as stage I (7.14%), 3 as stage II (21.43%), 3 as stage III (21.43%), and 7 as stage IV (50%). In cases with stages I and II, the “wait and see” policy was applied. In cases with stages III and IV, elective neck dissection is generally recommended. However, at the hospital where the study was conducted, the relatively small size of the tumor and the feasibility of primary closure were considered. Therefore, the surgical approach adopted was minimally invasive, opting not to perform neck dissection. The chi-square test through cross-analysis revealed a significant correlation between pTNM stage and incidence of DNM (*p* = 0.03).

Patients who had relatively large size of tumors compared to other patients or who revealed positive and close resection margins on biopsy results underwent postoperative radiation therapy. The presence of postoperative radiation therapy showed a significant correlation with DNM (*p* = 0.008). The results of histopathological examination in patients with DNM showed that among the patients, well differentiated consisted of 4 cases, moderate differentiated of 10 cases, and none of the cases was identified as poorly differentiated. Histologic grade did not show significant associations with DNM. In the case of the depth of invasion (DOI), the average was 7.85 ± 5.48 mm (range, 1 to 23 mm). DOI refers to the degree of penetration into tissues beneath the epithelial surface. In our study, we categorized the cases into two groups based on the DOI: those with DOI less than 5 mm and those with 5 mm or greater. We conducted a cross-analysis to investigate the relationship between DOI and the occurrence of DNM and confirmed a significant correlation (*p* = 0.0085).

Among patients with cN0, the primary site of tumor was most frequently observed in the tongue (*n* = 15), followed by maxillary gingiva (*n* = 12), mandibular gingiva (*n* = 10), buccal mucosa (*n* = 7), retromolartrigone (*n* = 6), floor of mouth (*n* = 3), lip (*n* = 1), mandibular vestibule (*n* = 1), and soft palate (*n* = 1). Especially in the case of patients with DNM, the tongue (*n* = 5), followed by maxillary gingiva (*n* = 3), floor of the mouth (*n* = 2), buccal mucosa (*n* = 2), mandibular gingiva (*n* = 1), and retromolartrigone (*n* = 1), the primary tumor sites were not significantly associated with DNM.

**Fig. 2:**
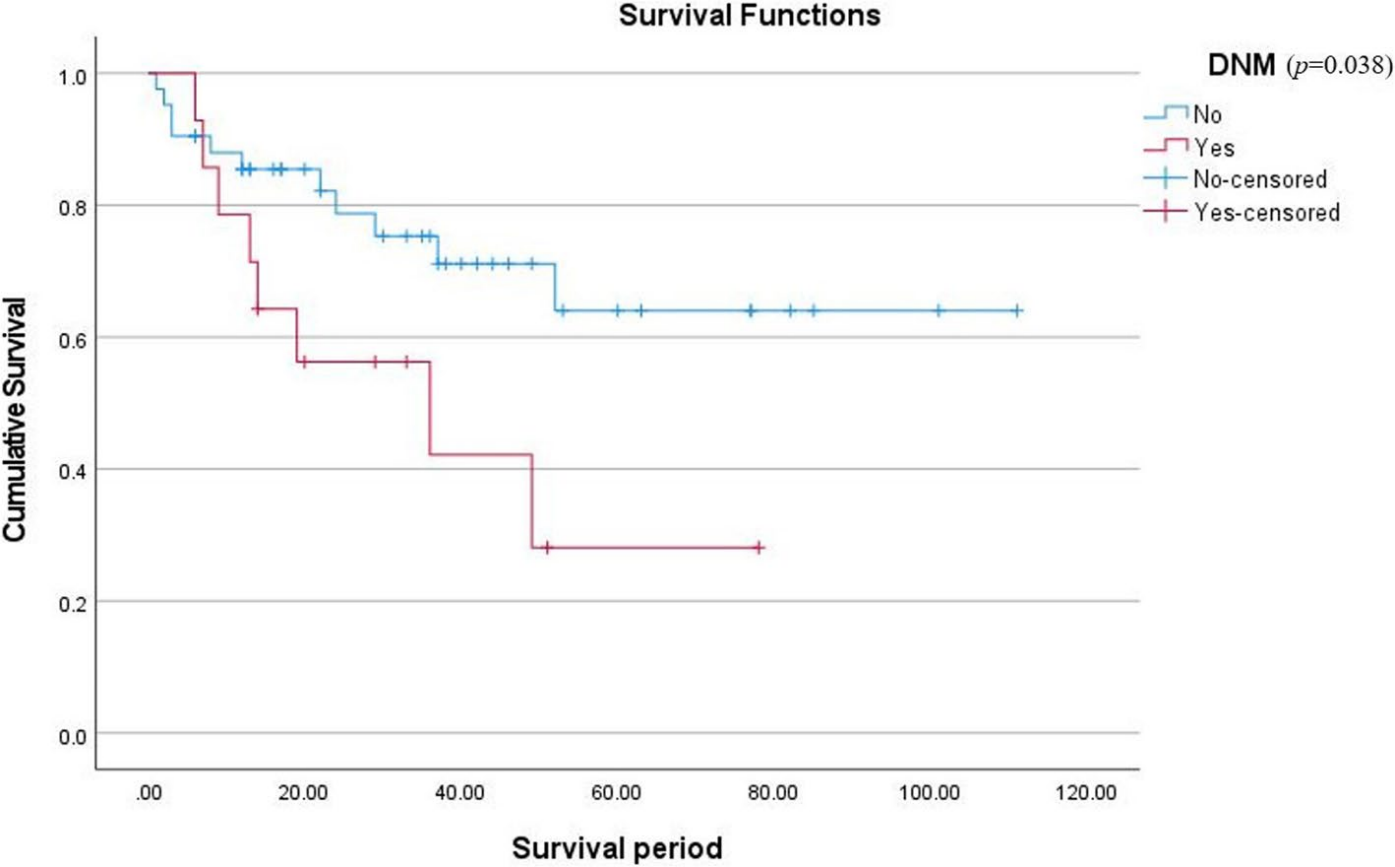
Comparison of cumulative survival rates between patients with incidence of delayed neck metastasis and without delayed neck metastasis (disease-free)

**Fig. 3:**
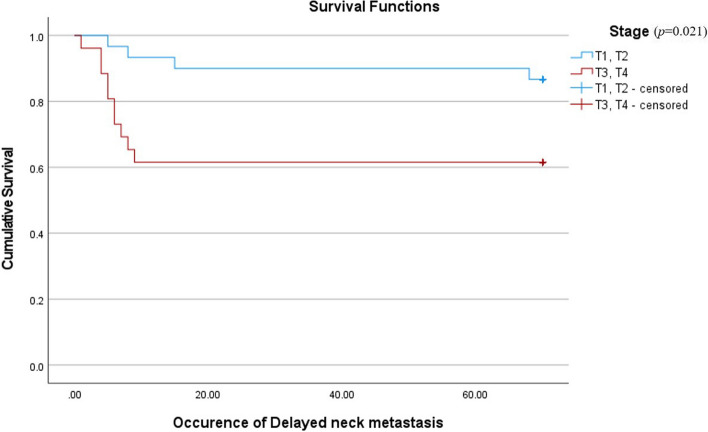
Occurrence of delayed neck metastasis according to pTNM stage

**Fig. 4:**
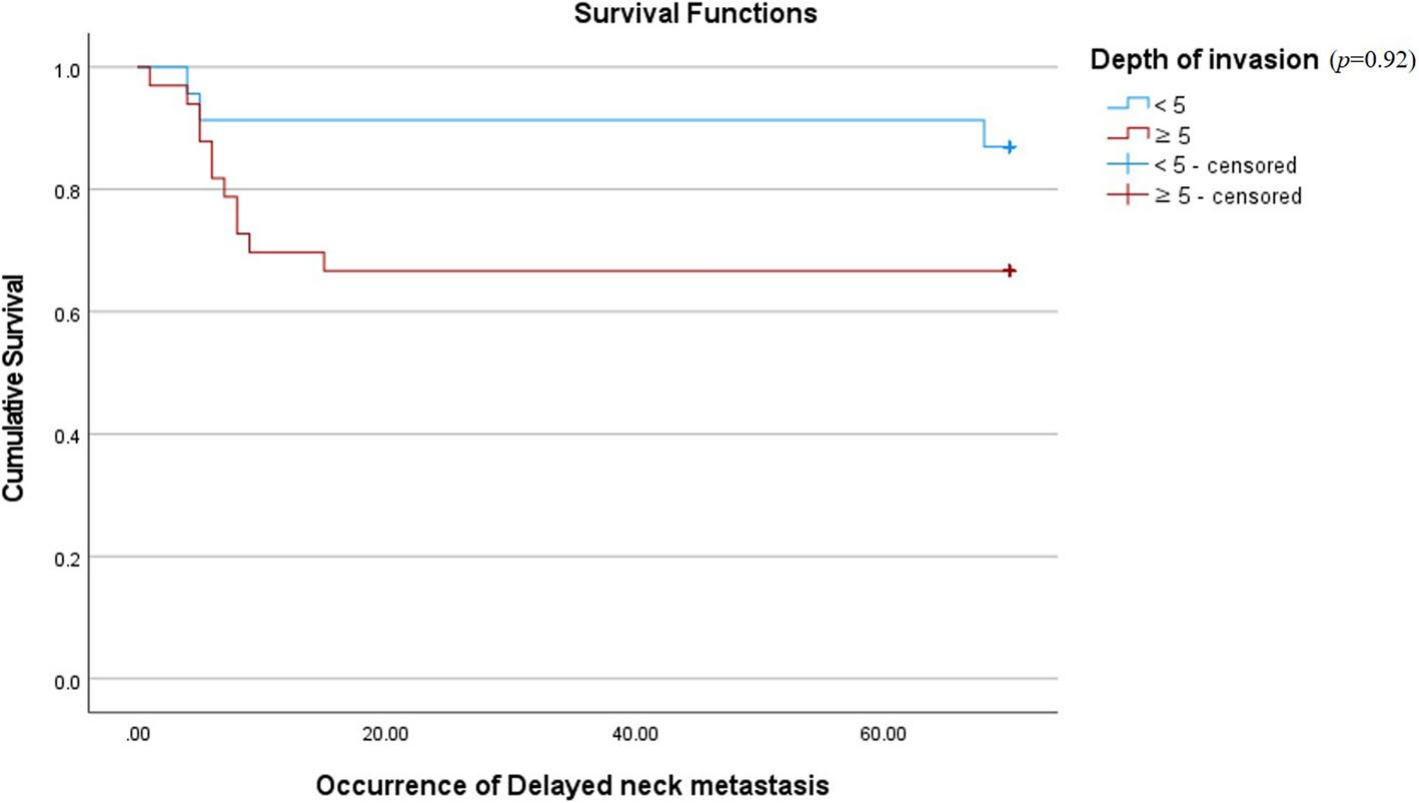
Occurrence of delayed neck metastasis according to depth of invasion

**Fig. 5:**
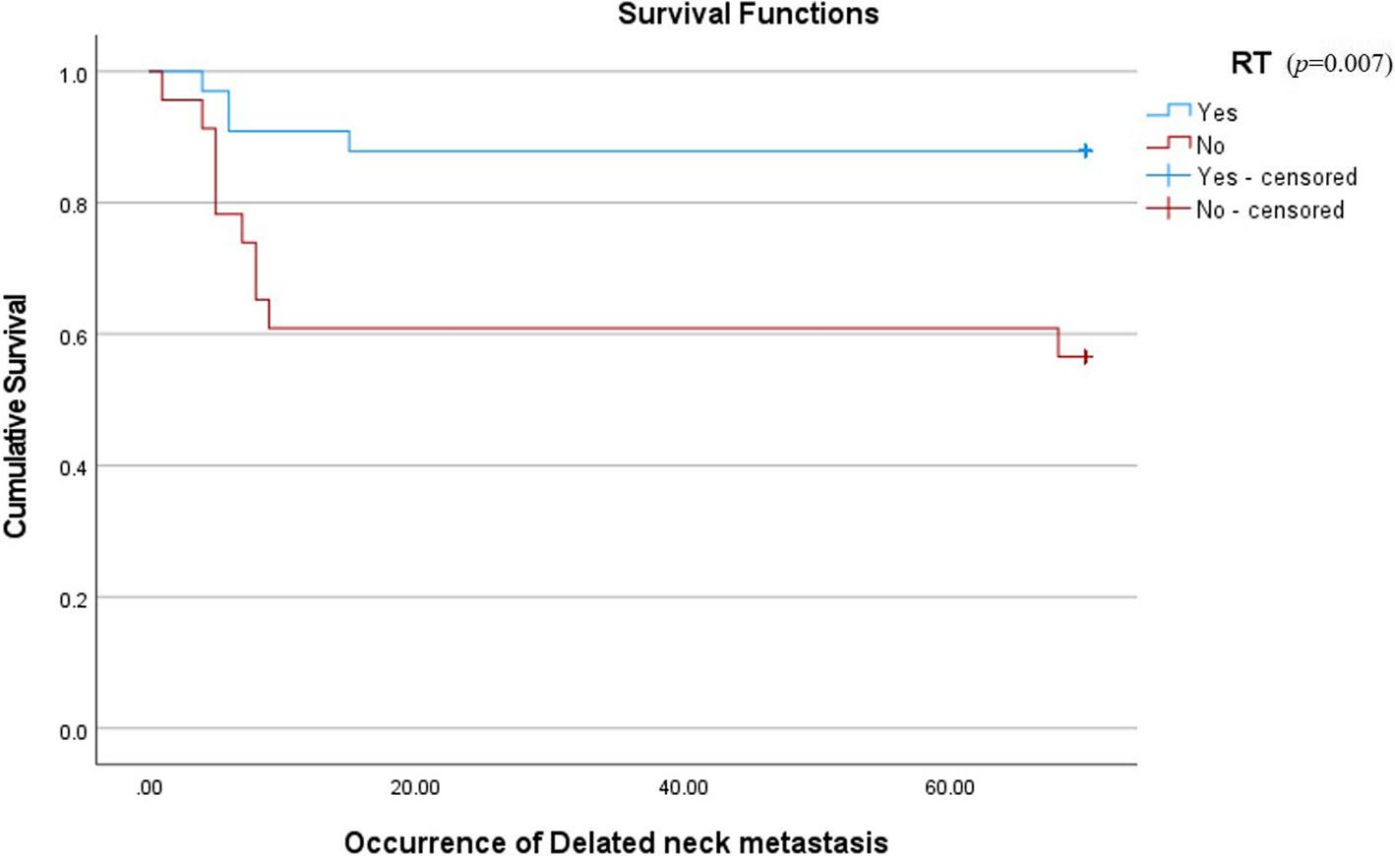
Occurrence of delayed neck metastasis according to radiation therapy

Considering prognostic factors that have shown a significant relationship with neck metastasis, this study compared the cumulative survival rate (CSR) between patients with an incidence of DNM after primary surgery and patients who were disease-free. Also, to confirm the correlation between the prognostic factors and DNM, a Cox proportional hazard model analysis was performed. Patients who had DNM seemed to be showing poor overall prognosis and survival rates (Fig. 2). This evaluation and differences in CSR were confirmed by the log rank test (*p* = 0.038). The result of the Cox proportional hazard model showed that pTNM stage was the most significant covariant of the incidence of DNM among three major prognostic factors (*p* = 0.04) (Fig. 3, Table 3). Also, to evaluate the relevance of factors which turned out to be related to the occurrence of DNM, Kaplan–Meier curves showing the occurrence of the DNM were provided (Figs. 3, 4, and 5).

**Table 3:** Overall test table for model coefficients in the Cox proportional hazard model including significant prognostic factors of delayed neck metastasis

	Overall (score)			Change from the previous step			Change from the previous block		
− 2 log likelihood	Chi-square	df	Sig	Chi-square	df	Sig	Chi-square	df	Sig
79.618	13.222	3	.004	13.198	3	.004	13.198	3	.004

## Discussion

DNM after oral cancer is a rare clinical phenomenon which the etiology has not been clearly identified. There are some literature and reviews about the event followed by OSCC of the tongue [5, 6, 8, 17, 18]. However, only few research was related to OSCC at other sites [9, 10]. According to previous studies, it is clear that neck metastasis is the most significant predictor of prognosis in cases of OSCC [11, 12]. The presence of neck metastasis results in approximately 50% reduction of the cure rate [23]. In consequence, the disease can significantly reduce the survival rate by more than 50% [22].

This study, conducted as a retrospective investigation into a highly rare phenomenon, holds clinical significance as it involved long-term tracking and analysis of patient data. Predicting the occurrence of DNM after surgery for OSCC is challenging, as it involves various host and tumor factors. Analyzing these factors is important for understanding the causes of DNM, which, in turn, plays a pivotal role in predicting patient prognosis and establishing appropriate treatment strategies. This study contains cumulative patient data of 17 years since 2006 to precisely analyze the relatively unreported field of DNM. Statistical analysis was based on demographic characteristics, patient factors, and tumor factors to estimate the prognosis of the disease.

Previous studies have suggested several patient and tumor factors related to the prognosis of OSCC. Some studies researched occult neck metastasis of node-negative cases [13, 24, 25]. Others suggested the risk factors and compared the survival rate of the patients [22]. Still, others have reported neck metastasis of the contralateral side associated with the risk factors [9, 10]. Such factors include demographic characteristics, size, pTNM stage, regional metastasis, neck dissection, depth of invasion, primary tumor site, clinical stage, and postoperative radiation therapy. However, DNM has not been reported frequently. Also, studies analyzing a direct correlation between risk factors and DNM and estimating survival rates based on significant factors were rare. Our findings did not reveal any significant difference in the occurrence of DNM concerning age, sex, distant metastasis, histopathological differentiation, perineural invasion, and lymphovascular invasion. Three factors that showed a significant correlation with DNM were pTNM stage, depth of invasion, and postoperative radiation therapy. A recent study has shown that the primary location of the tumor and pTNM stage are important predictors of neck metastasis [24, 25]. In case of survival, several studies have suggested that the pTNM stage at the time of diagnosis turned out to be a crucial factor [22, 26]. In our study, cumulative survival rates of patients with DNM and without DNM were estimated.

When comparing the CSR of patients who had DNM after the initial surgery and patients who remained disease-free, the latter group showed a better prognosis in survival. Also, the most important covariant related to the incidence of DNM turned out to be pTNM stage by the result of the Cox proportional hazard model. Although the result of this analysis indicates the significance of pTNM as an important factor contributing to the occurrence of DNM, it may not reveal how much more likely DNM is to occur in advanced stages compared to early stages. However, the result can imply the assertion made in previous studies, which indicates that as the pTNM stage advances, the likelihood of survival decreases [27, 28]. This implies that even in cases of DNM after primary surgery, the lower pTNM stage indicates a greater chance of successful treatment in OSCC. Also, the higher CSR of disease-free patients suggests that the recurrence of neck lymph nodes may be indicative of a higher cancer malignancy or a more rapid disease progression. Therefore, it can be said that rapid detection of recurrence is crucial for the prognosis of DNM.

In our study, the *χ*^2^ test has shown a significant correlation between DNM and depth of invasion. This result is coherent with previous studies that suggest the anatomical DOI is associated with nodal metastasis and reported to be a predictable factor of neck metastasis [29–33]. This factor can be predicted to be related to the pTNM stage mentioned above, because a malignant tumor that reveals higher DOI is likely to classify into a higher level of pTNM stage, in terms of either depth or size. However, when the DOI was evaluated considering the cumulative survival rate, it did not show a significant correlation with the occurrence of DNM in this study. This is speculated due to the small sample size, since numerous previous studies have verified the significance of the factor, indicating the need for further evaluation in future research.

Postoperative radiation therapy implies a positive or close margin in biopsy results. According to the treatment protocol, adjuvant therapy seems to be less effective due to the potential for localized or regional invasion in OSCC [22]. In this study, cross-analysis results revealed a higher tendency for patients undergoing radiation therapy to experience DNM. It is considered that when radiation therapy is administered due to the result of close margin after initial surgery or the high malignancy of the tumor, the likelihood of metastasis to other areas is increased. Therefore, it is believed that the obtained results are attributed to the elevated probability of DNM in such cases.

In order to explain the presence of DNM, the theory of field cancerization is considered. Field cancerization was first suggested in 1953 while pathologic tissue was found in clinically normal tissue around OSCC [34]. The findings led to a conclusion that the normal mucosa near OSCC has gone through changes in its characteristics due to exposure to carcinogens accelerating multiple foci development of malignant transformation [35]. This could be explained by molecular and genetic tissue alterations [36–40]. The nearby healthy mucosa exposed to carcinogens can also undergo abnormal molecular changes. The molecular alterations, identified as key signs of field cancerization, involve mutations in oncogenes and tumor suppressor genes, loss of heterozygosity, and genomic instability [37, 38]. Cells carrying these modifications are known to gain the capability to initiate and expand the pre-cancerous field. It is theorized that these altered, pre-cancerous cells may eventually replace the normal mucosal cells, making the epithelium more susceptible to further genetic alterations, thereby triggering the formation of tumors. This suggests that if a tumor arises from tissues which are altered by field cancerization, there is an increased likelihood of cancer cells spreading through the lymphatic vessels to nearby lymph nodes. This signifies that mutations are occurring over a wide area even before the tumor is detected. Regarding this theory, pre-surgical examinations such as sentinel node biopsy should be performed to identify entrapped tumor-suppressing gene or oncogene [41]. The sentinel node is known to be the first group of lymph nodes in a regional lymphatic basin where cancer is likely to spread from the primary tumor site. Therefore, this procedure can determine the extent of cancer involvement and guide treatment decisions.

The limitations of this study include a relatively small sample size and a short follow-up period for patients who underwent initial surgery recently. Especially for patients who underwent surgery relatively recently, DNM can potentially occur at any time in the future. Additionally, there is a limitation in not considering cases such as local recurrence and second primary SCC, which could be directly related to neck failure. However, in the obtained sample for this study, the number of such cases was very limited, making it challenging to incorporate them into the statistical analysis. It is deemed essential to address these aspects in future research.

## Conclusion

Our research identified several prognostic factors that exhibit a significant correlation with the likelihood of DNM, offering valuable insights for predicting this occurrence and assessing prognosis. Also, by analyzing and comparing the CSR of patients who experienced DNM and who did not after surgery, the results found by the study can contribute to the prediction of prognosis of the treatment.

## Data Availability

Not applicable.
